# Cardiac magnetic resonance imaging in Alström syndrome

**DOI:** 10.1186/1750-1172-4-14

**Published:** 2009-06-10

**Authors:** Margaret A Loudon, Nicholas G Bellenger, Catherine M Carey, Richard B Paisey

**Affiliations:** 1 South Devon NHS Trust, Torbay Hospital, Newton Rd, Torquay, TQ2 7AA, UK; 2 Royal Devon and Exeter Foundation Trust, Royal Devon & Exeter Hospital, Barrack Road, Exeter, EX2 5DW, UK

## Abstract

**Background:**

A case series of the cardiac magnetic resonance imaging findings in seven adult Alström patients.

**Methods:**

Seven patients from the National Specialist Commissioning Group Centre for Alström Disease, Torbay, England, UK, completed the cardiac magnetic resonance imaging protocol to assess cardiac structure and function in Alström cardiomyopathy.

**Results:**

All patients had some degree of left and right ventricular dysfunction. Patchy mid wall gadolinium delayed enhancement was demonstrated, suggesting an underlying fibrotic process. Some degree of cardiomyopathy was universal. No evidence of myocardial infarction or fatty infiltration was demonstrated, but coronary artery disease cannot be completely excluded. Repeat scanning after 18 months in one subject showed progression of fibrosis and decreased left ventricular function.

**Conclusion:**

Adult Alström cardiomyopathy appears to be a fibrotic process causing impairment of both ventricles. Serial cardiac magnetic resonance scanning has helped clarify the underlying disease progression and responses to treatment. Confirmation of significant mutations in the *ALMS1 *gene should lead to advice to screen the subject for cardiomyopathy, and metabolic disorders.

## Introduction

Alström syndrome is an autosomal recessive disorder caused by mutations in the *ALMS1 *gene (*OMIM 203800*) and is known to affect over 450 sufferers worldwide [1,2]. The gene is located on chromosome 2p13 and codes for a protein linked with the centrosome, though its precise function is unknown [3,4]. Many different mutations of the gene have been described but so far numbers have been too small to uncover genotype-phenotype correlations [5]. The diagnosis should be considered by ophthalmologists and geneticists in children with early onset retinal dystrophy, particularly when accompanied by any other feature of the syndrome such as insulin resistance and obesity, sensorineural hearing loss, or cardiomyopathy in infancy or adolescence [2]. Other features which may present later include short stature, chronic renal and hepatic dysfunction, bladder instability, early onset type 2 diabetes, and secondary hypothyroidism, hypogonadism in males, hypertriglyceridaemia, and kyphoscoliosis. The wide range of possible comorbidities in Alström subjects can reduce quality and length of life. Early diagnosis will afford the opportunity to introduce effective therapies for heart failure, diabetes, renal impairment, and hyperlipidaemia. This is particularly true of cardiomyopathy, which presents acutely in childhood in 45% with potentially fatal results if unrecognised, and recurs or develops de novo in 65% in adolescence [6].

The aetiology and pathophysiology of cardiomyopathy in Alström are incompletely understood. Echo studies have reported dilated cardiomyopathy [7] while a smaller study incorporating tissue Doppler imaging (TDI) found a restrictive pattern [8]. A small autopsy series of 5 patients demonstrated myocardial fibrosis. [6]

Early diagnosis of cardiac impairment with imaging has been problematic. There are inherent technical difficulties with echocardiographic scanning in Alström patients due to characteristic body habitus with markedly increased subcutaneous body fat, kyphoscoliosis and pulmonary disease[9,10]. Echocardiography lacks the inter-study reproducibility to enable early detection of left ventricular changes in an individual. The assessment of the impact of this syndrome on the right ventricle is severely limited by the inability of echocardiographic scaaning to adequately visualize the right ventricle. Other techniques, such as MUGA and PET nuclear imaging, provide poor spatial resolution and incur significant radiation exposure in young patients requiring serial studies. Cardiac magnetic resonance imaging (CMR) offers the opportunity to acquire high temporal resolution movie images in any desired plane independent of body habitus and free of radiation. CMR imaging allows the detection of sub-endocardial, mid wall or epicardial processes. The high reproducibility enables subtle changes to be detected in serial studies and offers the promise of tracking the natural history and investigating the influence of interventions [11-14]. We describe the first CMR analysis of a series of Alström patients.

## Methods

### Participants

Torbay District General Hospital, South Devon NHS Trust, is the National Specialised Commissioning Group centre for Alström syndrome. The CMR scans were clinically indicated in 7 of the 25 Alström subjects in the adult clinic to follow up echocardiographic abnormalities. They took place between 2007 and 2009 in the Royal Devon and Exeter Hospital NHS Foundation Trust.

Seven Alström patients (4 male, ages 19 to 43 years) underwent at least one cardiac magnetic resonance imaging scan. Three of the adults had cardiomyopathy in infancy, two of whom were receiving treatment for active adult onset cardiac failure. All were insulin resistant defined by a serum C peptide level of >1500 pmol/L, one hour after a high carbohydrate meal (Table 1).

**Table 1:** Characteristics of the Alström patients studied.

	Normal Values	1	2	3	4	5	6	7
Age yrs		37	24	43	19	39	26	23

Gender		M	F	M	F	M	F	M

BMI	<25	47	36	34	33	32	29	29

Genetically confirmed AS		heterozygote	yes	yes	yes	yes	yes	yes

Infantile cardiomyopathy		no	yes	no	no	yes	yes	no

Blood Pressure		134/56	90/60	112/72	110/56	145/90	100/64	132/81

eGFR		72	57	58	>90	35	44	>90

Ejection Fraction on CMR	>55%	61	25	40	60	59	55	50

Clinical failure in last 12 months		no	yes	no	no	no	yes	no

NTBNP ng/L	<150	24	108	65	14	279	495	6

Triglycerides mmol/L	<1.5	7.3	2.6	8.8	2.3	4.0	4.0	1.95

Chol:HDL		5.4	5.6	8.3	4.3	4.6	8.5	6.72

HBA1C %	4.0–6.1	11.0	8.5	9.4	5.2	16.8	6.2	5.2

Thyroid and gonadal function were corrected and stable for at least 3 months at the time of scanning. All received metformin but not glitazones and their usual cardiac medications. Chronic kidney disease stage 1 or 2 was present in 5 of the subjects.

-Quoted blood results and blood pressure are taken from the quarterly clinic prior to the CMR scan.

-Due to the visual and hearing sensory deficit these patients experience, each patient was coached in breath holding in response to tactile cues.

### CMR imaging protocol

All patients underwent CMR using a Siemens Avanto 1.5 T scanner with a cardiac coil. Static transaxial black blood (HASTE) images were acquired as well as T1 and T2 weighted thin transaxial slices, with and without fat saturation. Left ventricular function was assessed by acquiring a stack of short axis cine images as previously described [15,16]. Diastolic function was assessed by acquiring through plane velocity encoded images at the level of the mitral valve. First pass perfusion images were performed in three planes using 0.05 mcg/kg of gadolinium (Magnevist; Schering-Bayer). An additional dose of Gadolinium was given to provide a total of 0.2 mmol/kg and short and long axis late enhancement images acquired after 15 mins using a 2D TurboFLASH (fast low angled shot) sequence (8 mm slice thickness, 2 mm gap, α = 23°, trigger 2 (unless bradycardic when trigger 1 used), number of segments 25, the TI adjusted for optimal nulling, 250 to 350 ms) [17]. Where present, artefacts were excluded using phase swaps and/or long axis cuts. A whole ventricle 3D turboFLASH sequence was also used as a back-up. Transaxial slices were acquired through the liver, both in and out of phase to document fatty infiltration.

### Data Values

Normal values were adjusted for age, gender and body surface area, using the standard reference values established by the Royal Brompton Hospital [18]. 95% Confidence intervals are quoted for 20–29 yrs as no data available for ages than 20 yrs.

## Results

All 7 patients successfully completed the protocol.

TABLE 2: Cardiac MR results from the Alström patient series.

### Anatomical imaging

**Figure 1:**
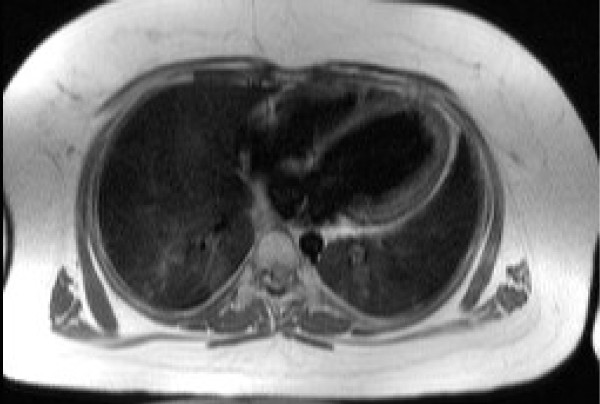
**Transaxial image of the thorax at the level of the heart illustrating the typical degree of sub-cutaneous and epicardial adipose tissue; hence the difficulty in imaging by echo (Patient 1)**.

All patients displayed normal situs with normal gross architecture of the heart and great vessels. There was a marked increase in adipose tissue in all cases. (figure 1)

### Morphological imaging

**Figure 2:**
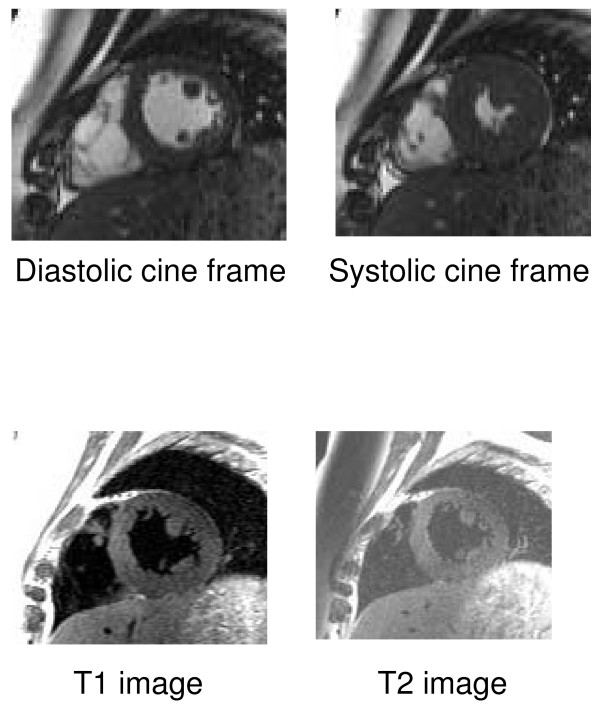
**Examples of different sequences for the same short axis slice**.

No patients displayed intra-myocardial high signal on T1, T2 stir images (figure 2) There was obvious high signal epicardial fat deposition that was appropriately nulled on fat saturation imaging, but no infiltration of the left or right heart myocardium. This would suggest there was no fluid or fatty infiltration of the myocardium. There were no areas consistent with calcium infiltration of the myocardium.

### Functional imaging

A spectrum of functional abnormalities was noted, with 4 of the seven patients having dilated left ventricular end systolic volumes. All patients had some degree of left ventricular dysfunction and 5 patients displayed right ventricular dysfunction. (Additional file 1). There was a mild reduction in left ventricular myocardial mass in most patients, when corrected for body surface area.

Specific segmental wall motion analysis revealed systolic regional wall motion abnormalities even in those patients with preserved left ventricular volumes and reasonable function.(Additional file 1)

### Perfusion imaging

Patients displayed patchy mid-wall perfusion abnormalities that corresponded with areas of delayed enhancement following gadolinium. The sub-endocardium was noticeably spared. The perfusion abnormalities were most prevalent in the mid and basal septum which was independent of the thickness of the myocardium. Given the nature of the thin wall of the right ventricle it was not possible to accurately assess perfusion abnormalities in this territory.

### Delayed Enhancement imaging

**Figure 3:**
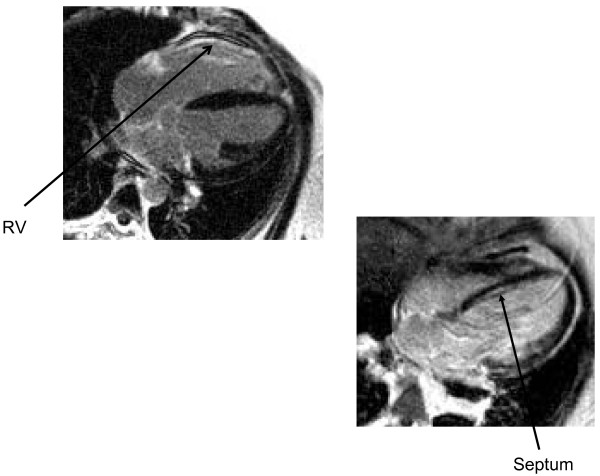
**Areas of hyper-enhancement following gadolinium**.

Four of the seven patients displayed some degree of mid wall late enhancement, with a preference for the basal lateral and mid septal walls. Interestingly, three of the seven patients also displayed some degree of right ventricular enhancement. (figure 3)

### Follow up studies

Two subjects had further studies one year later, subject 1 to assess effects of change in therapy, and subject 6 because of progression from NYHA heart failure grade 1 to 3.

**Figure 4:**
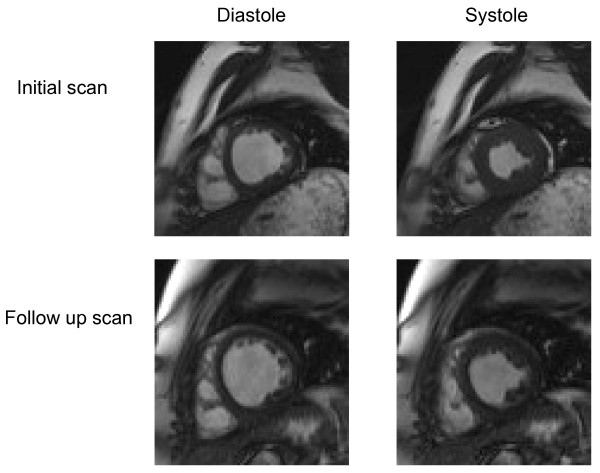
**Diastolic and systolic frames of one short axis cine image on initial and follow up scans, showing an increase in end diastolic and end systolic volume and reduced ejection fraction at follow up**.

In subject 1 all the cardiac MR parameters have remained unchanged. In subject 6 there was a striking increase in cardiac fibrosis and decrease in all parameters of cardiac function (figure 4). This has led to preparation for cardiac transplantation in subject 6.

### Liver fatty infiltration

There was a heterogeneous expression of diffuse fatty infiltration. One patient had marked segmental infiltration (Additional file 1, patient 2).

## Discussion

The frequent coexistence of obesity, insulin resistance, glucose intolerance, hypertension, dyslipidaemia and renal dysfunction in the syndrome [19] has led to the suggestion that these potent cardiovascular risk factors lead to early sub-endocardial infarction with progressive loss of myocardial tissue and function. Despite the presence of impaired ventricular function no patient had evidence of myocardial infarction. Due to the demanding nature of the protocol it was not felt appropriate at this stage to add adenosine stress perfusion. Although patchy rest perfusion defects were seen that correlate with areas of fibrosis, the lack of a formal stress test means that the presence of myocardial ischaemia was not assessed. Nevertheless, no patient displayed any sub-endocardial late enhancement suggestive of myocardial infarction and high-resolution late enhancement CMR allows small areas of sub-endocardial infarction to be visualised [20]. However our subjects are relatively young and the lack of stress testing means that coronary atheroma which might contribute to cardiac dysfunction cannot be excluded.

Five of the seven patients displayed mid wall hyper-enhancement of the left ventricle. Since gadolinium cannot enter live cells, the late enhancement represents late accumulation of gadolinium in areas of increased extra-cellular space, classically areas of apoptosis and fibrosis. The finding that all patients had some degree of cardiac fibrosis suggests that cardiac failure is related to degree of myocardial involvement. Subject 2 with clinically severe heart failure and most extensive CMR evidence of fibrosis had the highest HbA1c level, longest duration of diabetes (14 years), and persistent cardiac dysfunction from infancy.

In this study there was no suggestion of high signal fatty deposition in the myocardium despite obvious high signal epicardial fat, even in the patients suffering the worse cardiac function. This lack of myocardial fatty deposits occurred despite all but one of the patients displaying diffuse fatty infiltration of the liver.

A consequence of our findings is that co-morbidities in the syndrome and factors favouring progression to cardiac dysfunction, which might independently worsen cardiac function, should be sought and treated early. The combination of mixed dyslipidaemia, insulin resistance, diabetes, renal impairment and hypertension is very strongly linked to premature atherosclerosis, especially coronary artery disease in the general population. This set of disorders, known to occur frequently in Alström subjects should be assessed and treated energetically, even though coronary artery disease may not yet have developed in many of this young group of patients. Again the finding of significant mutations in the *ALMS1 *gene would encourage surveillance for coronary risk factors and treatment with hypolipidaemic, insulin sensitising and hypotensive therapies where indicated.

This is of importance to geneticists as continued search for phenotype-genotype correlations could lead to more accurate prognosis for Alström families if particular mutations were associated with more severe cardiac fibrosis. In addition, requests for genetic tests for the syndrome will come from a wide range of specialists including adult diabetologists, ophthalmologists, clinical geneticists, lipid experts and paediatric endocrinologists. Our results would support advice to referring clinicians to perform baseline cardiac MR on patients who screened positive for mutations in the *ALMS1 *gene.

Cardiomyopathy is a frequent cause of early mortality in Alström patients. While the syndrome remains incompletely understood, consistent findings were found throughout this patient group, which may give insights into the underlying cardiac pathology. There was no fluid or fatty infiltration of the myocardium in any patient and all displayed patchy left and right ventricular fibrosis and impaired left and right ventricular function, to varying degrees. Cardiac magnetic resonance imaging not only provides pathological insights, but gives the opportunity to detect early functional changes, track the natural history and progression of the disease, and assess the influence of therapeutic interventions, as well as guide referral for transplantation in this challenging group.

It seems likely that any cardiac pathological process in Alström patients will occur at the microscopic and micro-circulatory level in a diffuse pattern. Detecting such changes, even using CMR, is extremely challenging and many of the features we have described are subtle. As technology advances and future patients have serial CMR scans, more information on pathophysiology should emerge.

## Conclusion

This study describes the first results of cardiac magnetic resonance imaging in this rare population. Confirmation of *ALMS1 *gene mutations in a patient is a strong indication for cardiac magnetic resonance imaging to be performed and for treatment of risk factors for coronary atherosclerosis.

## Ethical Approval

South Devon Healthcare Trust ethics committee gave consent for systematic assessment of cardiac and vascular status in Alström subjects in 2007.

## Abbreviations

BSA: body surface area; LVEDVI: left ventricular end diastolic volume index; LVESVI: left ventricular end systolic volume index; LV Mass I: left ventricular mass index; RVEDVI: right ventricular end diastolic volume index; RVESVI: right ventricular end systolic volume index; LVEF: left ventricular ejection fraction; RVEF: right ventricular ejection fraction; WMA: wall motion abnormality; AS: Alström syndrome; CMR: cardiac magnetic resonance imaging; OMIM: Online Mendelian Inheritance in Man.

## Competing interests

The authors declare that they have no competing interests.

## Authors' contributions

ML drafted the manuscript, NB devised the magnetic resonance protocol, analysed and reported the scans, CM devised the study, RP devised the study and participated in the drafting of the manuscript. All authors read and approved the final manuscript.

## Supplementary Material

Additional file 1 **Table 2**. Cardiac MR results from the Alström patient series. Click here for file
